# Clinician and service user perceptions of implementing contingency management: A focus group study

**DOI:** 10.1016/j.drugalcdep.2011.05.016

**Published:** 2011-12-01

**Authors:** J.M.A. Sinclair, A. Burton, R. Ashcroft, S. Priebe

**Affiliations:** a University of Southampton School of Medicine, Southampton SO14 3DT, UK; b Queen Mary University of London, Unit for Social and Community Psychiatry, Newham Centre for Mental Health, London E13 8SP, UK; c Queen Mary University of London, London E1 4NS, UK

**Keywords:** Qualitative research, Contingency management, Applied and professional ethics, Behaviour modification, Substance misuse

## Abstract

**Background:**

Contingency management (CM), despite the evidence base for its effectiveness, remains controversial, with sub-optimal implementation. In 2007, UK guidelines recommended the use of CM in publicly funded services, but uptake has also been minimal. Previous surveys of service providers suggest differences in opinions about CM, but to date there has been no published involvement of service users in this debate.

**Method:**

Focus group methodology was used to explore systematically the attitudes, concerns and opinions of staff and service users about the use of CM, in publicly funded substance misuse services, to identify the key areas that may be influential in terms of implementation and outcome. Data were analysed thematically using the constant comparative method.

**Results:**

70 staff and service users participated in 9 focus groups. 15 themes of discussion around CM were identified, grouped into four categories: how CM was aligned to the philosophy of substance misuse services; the practicalities of implementation; wider ethical concerns; and how participants perceived the evidence for effectiveness.

**Conclusions:**

Robust process evaluation in different treatment systems is needed to define the active components of CM for implementation. Involvement of service users in this process is essential and is likely to provide valuable insights into the mechanism of action of CM and its effectiveness and uptake within complex treatment systems.

## 1 Introduction

Contingency management (CM) is the term for a range of behavioural interventions in which tangible positive rewards are provided to individuals contingent upon objective evidence of behavioural change. There is a well established evidence base (primarily from US treatment centres) for the effectiveness of CM as part of a treatment package for people with substance use disorders (Dutra et al., 2008; Plebani Lussier et al., 2006; Prendergast et al., 2006).

However, specific differences between UK and US health and welfare systems mean that there is likely to be significant differences in the cost-effectiveness of CM interventions depending on whether a service user, provider or societal perspective is taken. Within the UK, health and social care is financed through general taxation to provide universal coverage, which is free at the point of delivery to the patient. This means that the benefits of CM are most likely to be found at a societal perspective, as indeed has been the case with other substance misuse programme (Gossop et al., 2001). In the US, where most of the CM research has been undertaken (Dutra et al., 2008; Pilling et al., 2007) differences in incremental cost effectiveness ratios (ICERs) even between individual sites in multicentre research programmes suggest that treatment delivery factors and variability in patient groups may make a real difference to the cost-effectiveness of CM at an individual and provider level (Olmstead et al., 2007).

Surveys of treatment providers in the US (Benishek et al., 2010; Kirby et al., 2006; McGovern et al., 2004) and a qualitative study from Australia (Cameron and Ritter, 2007) show that a number of factors influence practitioner attitudes to CM, and their likelihood of adopting it as a treatment. These include practitioner understanding of the evidence base, the practicalities of implementing it, as well as the socio-demographic characteristics of the practitioners themselves, and how these might differ within teams, and between practitioners and management (Kirby et al., 2006). The effectiveness of a single behavioural intervention for any chronic medical condition including addictions is likely to be affected by multiple contextual factors including national health policies, funding priorities, individual and institutional views on the role of the state, and the responsibility of the individual in modifying behaviour. To date there have been no published studies from the UK of practitioners’ or service users’ understanding of and attitudes towards CM within the UK substance misuse services, which may further illuminate the processes involved in the uptake of complex and contentious interventions of this kind across different health systems.

In the UK, the national treatment guidelines on psychosocial interventions for drug misuse (National Institute for Health and Clinical Excellence, 2007) recommended the introduction of CM into UK drug treatment services, based on the international evidence, although recognising the paucity of evidence within the UK. Training of clinicians and improving public understanding of the benefits of using CM in substance misuse services were seen as important and necessary steps to be overcome for effective implementation to occur (Pilling et al., 2007). Other than a number of ‘demonstration sites’ that have not published their findings, there has been no systematic implementation of CM in the UK.

The aim of this study was to explore systematically the attitudes, concerns and opinions of staff and service users about the use of CM, as detailed by National Guidelines (Department of Health, 2007; National Institute for Health and Clinical Excellence, 2007), in publicly funded substance misuse services (see Table 1). As there is no previous published data in this area, qualitative methods (focus groups) (Kitzinger, 1995) were used to define key areas, and allow for the identification of factors and processes that may be influential in terms of implementation and outcome.

## 2 Method

### 2.1 Participants and procedures

Focus groups were conducted to explore participant attitudes and opinions about the implementation of CM. Purposive sampling was used to include key stakeholders using and working in and with publicly-funded specialist substance misuse services. Staff and service users from specialist substance misuse services were recruited to one of nine focus groups.

Specialist addiction psychiatrists were identified through attendance at one of two Specialist Clinical Addiction Network (SCAN) conferences. An information sheet was sent out to delegates before each conference to invite them to take part in a focus group. For the recruitment of other staff, we approached four specialist substance misuse teams, two within East London (an area of high urban deprivation) and two within Hampshire, a mixed rural and urban area. Recruitment of staff was through the team manager. Service users were approached by staff and/or team managers working in specialist substance misuse services to participate in one of two focus groups, whilst a third group were recruited through their links with a voluntary service user advocacy group.

### 2.2 Data collection

**Fig. 1:**
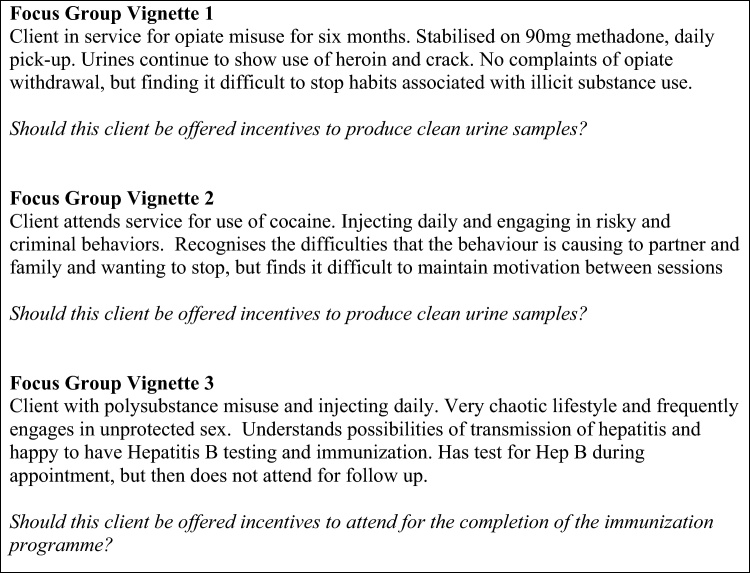
Case vignettes used to stimulate focus group discussion.

Focus groups were conducted between May 2008 and 2009. Each group lasted approximately 1 h and consisted of between two and 12 people. All groups were conducted by a facilitator and co-facilitator, audio digitally recorded and transcribed verbatim. All followed the same procedure. After participants introduced themselves, the facilitator read a pre-written summary of the principles of CM as proposed by the NICE Guideline ‘Drug misuse: psychosocial interventions’ (National Institute for Health and Clinical Excellence, 2007). Participants were asked to discuss their initial thoughts on the guideline with each other for approximately 15 min, before being given three brief clinical vignettes to explore. Each vignette described a scenario for which recommendations were made in the NICE Guidelines (substitute prescribing for opiate dependence, cocaine misuse, completion of immunization programme for Hepatitis B). The vignettes are presented in Fig. 1. After each vignette was presented, the group were asked to discuss whether a client should be offered incentives in the given situation, and the reasoning behind their opinions.

### 2.3 Data analysis

Data collection and analysis occurred simultaneously using analytic techniques of the constant comparative method (Glaser, 1992; Glaser and Strauss, 1967). All transcripts were read and corrected by the facilitator and co-facilitator of each group, and annotated with field notes taken by the co-facilitator during the group, to ensure that the context of what was said, and other social cues, (e.g., laughter, murmured agreement, etc.) was retained. Transcripts and the associated annotations were imported into the qualitative software package NVivo7 (QSR International Pty Ltd., 2006) to aid analysis. Three of the researchers (JS, AB, SP) read the transcripts and independently defined a preliminary coding scheme which was discussed in the research team. The final coding scheme was generated by an iterative process as further data were collected until saturation was reached. Data were coded by AB independently reading the transcripts and coding all material using NVivo7 (QSR International Pty Ltd., 2006) software, with continuous comparison and discussion where discrepancies arose. The research team discussed and analysed the link between the early dense codes and broader themes to ensure conceptual clarity and consistency across the themes and further recoding where required.

## 3 Results

### 3.1 Participants

A total of nine focus groups were carried out, consisting of: current service users (2 groups: *N* = 2, *N* = 6), ex service users (1 group: *N* = 6), specialist addiction psychiatrists (2 groups: *N* = 9, *N* = 11) and multidisciplinary staff teams working in publicly-funded specialist substance misuse services (4 groups: *N* = 9, *N* = 7, *N* = 10, *N* = 10). Overall, there were 70 participants, including: 14 current or ex-service users (patients), 20 addiction psychiatrists, and 36 staff working in multi disciplinary specialist substance misuse teams. The sample captured a range of experience of staff with the mean length of service being 10 years (varying from 10 months to 41 years). Service users had been using substance misuse services for an average of 14 years (ranging from three to 36 years). Participants were aged between 22 and 62 (mean age 45 years old) and 66% of the sample was male.

### 3.2 Main themes emerging from focus groups

**Fig. 2:**
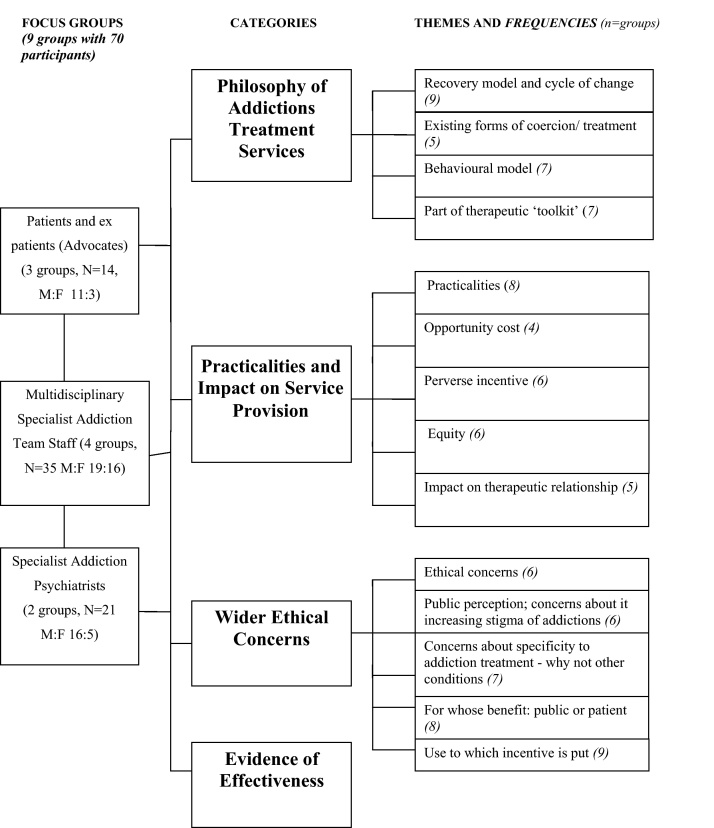
Focus group categories, emerging themes.

Although the different groups varied substantially in their composition, which resulted in different levels of discourse, and focus around the subject, there was a high level of saturation across the groups in terms of the themes which emerged. In most areas there were substantially similar views expressed by the professional and service users; areas of disagreement or different emphasis are highlighted where these occurred. The final agreed coding scheme contained a total of 40 codes which were applied consistently across all the group transcripts. On review of the content of these codes, they could then be consolidated into 15 main themes, presented here under four broad categories (see Fig. 2), with illustrative examples given in Table 2.

### 3.3 Philosophy of substance misuses treatment and services (see Table 2.1 for examples)

All 9 of the groups spent some time discussing the role of CM and where it ‘fitted’ within current treatment principles. Concern was expressed from the professional groups that it reintroduced a more ‘paternalistic’ approach to treatment that had been moved away from in recent years in the UK. Discussion on the potential impact it might have on damaging any intrinsic motivation of the service user to engage with treatment was a particular feature from those who were now themselves ‘in recovery.’ There was also much debate about how the focus of many services is currently on ‘harm minimisation’ (e.g., needle exchange, immunisation, maintenance substitute prescribing, etc.) rather than on abstinence and recovery. Consequently, the aim of CM to produce drug free urine (see vignette 1, Fig. 2) did not sit comfortably with this.

Seven groups acknowledged the powerful function of simple behavioural measures in shaping and changing behaviour in everyone's life, as well as the importance of ‘rewarding’ people, especially those with so little that was positive and reinforcing in their lives. However, the counter view was also proposed, that relatively small monetary values might reinforce peoples’ sense of lack of worth, whereas rewarding them with a non-monetary incentive tailored specifically to them (e.g., access to training, etc.) might be more beneficial. It is of interest that the two groups of service users currently within the treatment system did not discuss this aspect of CM.

There were differences across the groups as to whether CM as defined in the guidelines represented a form of coercion or whether it was a treatment incentive. Practitioners were divided as to whether take home methadone treatment was one of a range of evidence based therapeutic options available to the clinician (see below), or something more politically driven. If the latter, it fitted their broader concerns about the nature of society (e.g., seeing CM as part of a politically driven policy and the role of the state in peoples lives) which are part of their identity as a citizen, rather than as a health practitioner, and are therefore firmly held and not easily amenable to change (see Section 3.5 below).

As with the other aspects concerning the theories and mechanisms around CM, its use as one of the several therapeutic options was not really discussed by those still in treatment, but was a common and recurring theme across all other groups, particularly the ex-service user group. There was a general consensus that (if there was evidence of effectiveness), the use of CM *in principle* might be a useful addition to the therapeutic armamentarium. This idea was most positively endorsed by those in the professional groups with greatest experience and training. However, there was a range of views expressed: from unequivocal benefit, through to a more cautious acceptance of it. Concerns were raised that in a system with limited resources it may be seen as a cost saving alternative, replacing established and more valued interventions (e.g., time with a member of staff) and therefore best kept as a ‘last resort’.

Much of the discussions of aspects of treatment delivery are common to other aspects of health care where incentives are used as part of treatment. They were framed within the concepts of health economics and medical ethics and included the following five themes: practicalities of implementation; the opportunity costs of the intervention; the possibility of CM acting as a perverse incentive; issues of equity; and the potential impact on the therapeutic relationship (see Fig. 2 and Table 2.1).

The practicalities and potential problems of implementation was a major theme across all but one focus group (service users) and included aspects that would be anticipated from any discussion about change management. However, concerns were also expressed that were more specific to implementing a behavioural intervention, where it is well recognised that the precise details are integral to the effectiveness of the implementation, and the possibility of unintended consequences. Regarding the implementation of CM within a publicly funded system, participants in four groups (3 professional groups and the ex-service user group) expressed concerns about the opportunity cost of such a change of focus. All nine groups expressed concerns about the feasibility of the level of urine testing (three times per week). However, whilst the professional teams viewed this as being resource heavy and had concerns about the potential opportunity costs of delivery (see Table 2.1), the service user groups felt strongly that such a regime acted as a disincentive that would outweigh any benefit from the financial incentive offered.

Concerns about the notion of equity of access to interventions within the treatment system, and that CM might act to incentivise non-engagement (i.e., act as a perverse incentive) were discussed in 6/9 groups. Concerns about equity were primarily expressed in the professional groups. Service user groups felt it more appropriate for CM to be offered on an individual basis depending on the needs of the service user at a particular time, rather than being mandated to particular groups and certain points in their treatment journey. They specifically felt that it may be more helpful as an initial incentive to encourage people to attend services for assessment rather than once they were already engaged with services as suggested in the guidelines.

Finally, within this category was a discussion around the potential for CM to have a negative impact on the therapeutic relationship, as time could be spent reviewing (and arguing about) urine samples rather than discussing the treatment needs of the service user. Some of the multi-disciplinary team members expressed the view that acting as a ‘broker’ within a CM system where the result of a test had pre conditioned consequences ‘cheapened’ the work that they did. There was no mention in any group that implementation of CM *per se* might enhance the therapeutic relationship.

### 3.4 Wider ‘ethical concerns’ (see Table 2.3 for examples)

This category encompasses the broader, often less focussed and more abstract discussion that the groups held around the general concepts of using public money, within a health system that offers universal coverage, to incentivise people to change their behaviour. Frequently raised concerns across the groups included: whether the use of CM for people in substance misuse services further stigmatised this patient group within the public mind? Who was the real beneficiary of this kind of intervention – the service users themselves or the public at large? Was this policy being driven by political motivation rather than the evidence base? These discussions articulated concerns of moral principle and personal belief, which were not evidence dependent, were not changeable within the group discussion or remediable by research or policy clarifications.

One specific aspect mentioned by all 9 groups was the use to which any financial incentive might be put. All recognised the possibility that it might be misused to buy further drugs, and the ex service user group specifically mentioned how giving people ‘extra’ money at a vulnerable point in their treatment pathway may do more harm than good. Whilst issues of autonomy were mentioned, small financial incentives were seen as being specifically targeted at the poorer in society. Whilst any incentive could have a monetary value if traded, a common theme across the groups was that non-monetary incentives targeted to the person's particular need (e.g., funding for electricity, public transport) may be more beneficial.

The public versus personal benefit of CM was felt to be particularly relevant in the scenario where service users were incentivised to complete the full vaccination course for Hepatitis B (see Fig. 1, vignette 3). This was viewed more as a single ‘harm minimisation’ exercise that offered long term protection to others, and therefore with a clear objective and fixed outcome, rather than an as part of a more complex treatment intervention in its own right. Overall, even where participants had expressed concerns over using CM as a means of achieving abstinence in the earlier vignettes, they felt much more comfortable with this more circumscribed intervention that offered a tangible and sustained positive outcome to both the drug user and the wider society.

### 3.5 Evidence of effectiveness (9 groups)

There were three main sub-themes that were brought up by participants around CM pertaining to the relative importance of effectiveness of CM as an intervention. These were as follows (see Table 2.4 for examples):

1. A pragmatic approach that linked in with the discussions around having CM as part of a ‘tool kit’ of interventions. This could be summarised as ‘if it works, use it.’ This stance was primarily taken by the more experienced clinicians.
2. A critique of whether the evidence base (which was understood by participants to be primarily from USA treatment centres) may not necessarily work by the same mechanism or to the same effect within the UK health and social care system and that caution was required in assuming direct transposition.
3. A firmly held belief that even if there was evidence of effectiveness for an intervention, it did not mean that it was necessarily an acceptable addition to the treatment system. This was a theme from across the groups, generally held by a small number of people within each group, but expressed firmly and consistently, and not appearing amenable to change by the group process.

## 4 Discussion

The aims of this study were to explore systematically the attitudes, concerns and opinions of staff and service users about the use of CM in publicly funded substance misuse services and to identify the key areas that may be influential in terms of implementation and outcome. Below we summarise the findings and examine specifically what this study adds to the literature in terms of:

1. How CM may fit within the context of substance misuse programmes.
2. The concerns about implementation.
3. Broader concerns that might be seen to represent individuals’ beliefs and concerns as a citizen rather than that as patient or healthcare provider.

The causes of addictions are well recognised to be a complex interaction of biological, social and psychological factors and from a health perspective can be considered within a chronic disease model (McLellan et al., 2000), requiring a collaborative approach between professional and patient if long-term, sustained positive outcomes are to be achieved. Many substance misuse services in developed countries work within a multi-disciplinary, community treatment model. Consequently, the way that new interventions are viewed by clinicians (in their role as individual citizen as well as practitioner), and the collective philosophy of a treatment service will have a substantial impact on the effectiveness and cost-effectiveness of their implementation and uptake (Benishek et al., 2010; Cameron and Ritter, 2007; Kirby et al., 2006; McGovern et al., 2004).

### 4.1 What are the key aspects underpinning the discussion about the role of CM in substance misuse treatment programmes?

This study highlighted the issues most consistently discussed about the use of CM by service users (both current and past) and health professionals. The 15 different themes are concerns that will need to be considered in any evaluation of effectiveness of CM implementation within different clinical settings, and across different health care systems.

#### 4.1.1 Role of CM within the philosophy of substance misuse programmes

Whilst the evidence base from randomised controlled trials (RCTs) for the role of CM in substance misuse programmes is compelling (Dutra et al., 2008; National Institute for Health and Clinical Excellence, 2007; Pilling et al., 2007) the uptake into clinical practice has been less good (Kirby et al., 2006; Petry, 2006). The results of this study suggest that the overall aims of a treatment programme (e.g., whether the aim is for harm minimisation or abstinence) may be a significant factor in how a single intervention is viewed and the likelihood of its implementation. The methodology of an RCT, even of a complex intervention, specifically attempts to insulate the intervention under examination, from such contextual factors. This study suggests that it is a key concern for professionals that any single intervention is coherent with the broader goals of the service. In addition to this, these results add empirical data to suggestions from other authors (Petry, 2006; Pilling et al., 2007) that the wider societal and political values that practitioners and service users hold as citizens will have an impact on how new interventions are delivered and received within the healthcare system. Government policy and media coverage will also affect and be affected by societal trends at any particular time and have an impact on the perception and implementation of specific health policies (Reinhardt, 1990).

#### 4.1.2 Practicalities and impact on service provision

Whilst some of the discussion of the impact that implementing CM might have within the treatment system could be viewed as an anticipated response to implementing changes in any service and therefore amenable to good change management processes, there are specific details about CM that may impact on its implementation and effectiveness. Our results support the findings of Kirby et al. (2006), that staff have concerns about service costs associated with the schedule of urine tests required, and the need to target more positive treatment outcomes (e.g., improved health and wellbeing) than simply aiming for drug free urines. Our results also suggest that staff and service users felt that the schedule of urine testing was unreasonable and impractical, but could be ‘altered’ to make them more acceptable, whilst the evidence base (Griffith et al., 2000) shows that the frequency of tests is a core component of effectiveness. This demonstrates one potential mechanism for how effect sizes in clinical trials may have a different impact once they are adopted into routine practice.

Who should be offered CM was another consistent concern. The general consensus across the professionals was that it should be available to all service users at a particular point in the treatment system, to fulfil the principles of horizontal equity (providing equal healthcare to those with equal need) (Culyer, 1995) and to stop a system of perverse incentives being set up (i.e., service users being rewarded for non-adherence to treatment). There was also a concern that CM might potentially damage the therapeutic relationship. These are common concerns described in the literature about the use of financial incentives to change health behaviour across a range of conditions (Burton et al., 2010; Marteau et al., 2009; Oliver, 2009; Priebe et al., 2010), but one for which there is currently limited empirical data. However, the service user groups in our sample did not express any such concerns; in fact, all three groups discussed the importance of tailoring a specific incentive (financial or otherwise) only to those who might benefit from it, suggesting an understanding and acceptance of vertical equity (i.e., treating differently those who have different needs) (Culyer, 1995), as a key factor in ensuring CM was most effective. This service user perspective has not, as far as we are aware, previously been discussed in the literature and may have important implications in terms of how CM systems are implemented in practice.

#### 4.1.3 Broader concerns

There was much discussion within the professionals group about CM being considered as a politically driven initiative, which was extended to more general feelings of antipathy towards treatment guidelines. There is a substantial literature on the length of time that it takes to get new research adopted into practice (Benishek et al., 2010; McGovern et al., 2004). Although not all the groups were aware of the literature of the effectiveness of CM, there was a general assumption that a literature existed as the basis of a national guideline. However, as with other studies, practitioners were quick to cite that the research evidence did not reflect the complexity of the service users or clinical situation of routine practice and this affects the perception of its usefulness for clinical decision making (Miller, 1987; Greenhalgh et al., 2004; Kirby et al., 2006; Pilling et al., 2007).

### 4.2 Strengths and limitations

The study is limited by the relatively small number of participants who took part in the focus groups. Issues of generalisability have a different focus within qualitative work, in that a study of this kind seeks to raise awareness of the concepts and define the phenomena to be further refined and tested for prevalence using other methods (Craig et al., 2008). The smallest focus group only included two female service users (both working as prostitutes) but the relative privacy of this group allowed for an in-depth exploration of the issues that they may have been less happy to engage with in a larger group.

One of the strengths of this study is that the use of qualitative methods allows for a more in-depth and contextualised exploration of the factors which may influence the implementation and effectiveness of a complex intervention such as CM.

Previous studies have shown that there are differences in the attitudes of staff members to CM (Benishek et al., 2010; Kirby et al., 2006; McGovern et al., 2004; Petry, 2006) and this study highlights the complex interaction of professional attributes and personal beliefs that may underlie these attitudes. That many of the concerns about CM appear to be similar in this smaller number of UK practitioners to the larger US surveys (Benishek et al., 2010; McGovern et al., 2004) suggests the validity and generalisability of these results, and some common cross cultural themes that require more robust process evaluation in future RCTs. A final strength of this study is the inclusion of service users within the analysis, and the different emphasis that they bring to treatment decision making.

### 4.3 The role of process evaluation to help elucidate the core components of CM

There is a growing literature demonstrating the importance of including process evaluation as an essential part of clinical trials of complex interventions (Audrey et al., 2006; Craig et al., 2008; Hawe et al., 2004; Lewin et al., 2009). One suggestion is to standardise the process of the intervention rather than the components themselves (Hawe et al., 2004), thus intervention ‘integrity’ would be defined as the evidence of fit with the principles of the hypothesised change process (in this case CM) rather than trying to reproduce the ‘exact’ conditions in each site. In order to do this, the active ingredients of a complex intervention need to be defined, including delivery mechanisms (Craig et al., 2008). In psychological interventions the attitudes of both staff and patients towards the intervention and their perception of its place within the treatment system, are likely to be important active ingredients and need further elucidation.

### 4.4 Implications

CM has been shown to be an effective intervention in the treatment of substance misuse. However, it is controversial and uptake within treatment systems has not been as widespread as the evidence would warrant.

There is a need for robust process evaluation of CM in different treatment systems, to define the active components of the process and the mechanism by which they are working (Hawe et al., 2004). Involvement of service users and advocacy groups in this process is essential and is likely to provide valuable insights into the mechanism of action of CM as well as its effectiveness and uptake within complex treatment systems.

## Role of funding source

The authors were funded by the Wellcome Trust (grant reference: 081433/Z/06/Z). The funding body had no further role in the study design, in the collection, analysis and interpretation of the data, in the writing of the report or in the decision to submit the article for publication. All researchers were independent from the funding body.

## Ethical approval

The study was approved by East London and the City Research Ethics Committee 3 (07/H0705/81). The study was later extended to Hampshire Partnership NHS Trust services and ethical approval was given by Southampton and South West Hampshire Research Ethics Committee (A). Written informed consent was obtained from each participant in person before they took part in a focus group.

## Contributors

Authors JS, SP and RA designed the study and wrote the protocol. Authors JS, SP and AB undertook the data collection and analysis, and author JS wrote the first draft of the manuscript. All authors contributed to and have approved the final manuscript.

## Conflict of interest

No conflict declared.

## Figures and Tables

**Table 1:** Summary of the principles of Contingency Management from UK guidelines 2007.

Contingency management aimed at reducing illicit drug use for people receiving methadone maintenance treatment or who primarily misuse stimulants should be based on the following principles:
• The programme should offer incentives (usually vouchers that can be exchanged for goods or services of the service user's choice, or privileges such as take-home methadone doses) contingent on each presentation of a drug-negative test (for example, free from cocaine or non-prescribed opioids)
• The frequency of screening should be set at three tests per week for the first 3 weeks, two tests per week for the next 3 weeks, and one per week thereafter until stability is achieved
• If vouchers are used, they should have monetary values that start in the region of £2 and increase with each additional, continuous period of abstinence
• Urinalysis should be the preferred method of testing but oral fluid tests may be considered as an alternative

Ref.: National Institute for Health and Clinical Excellence (2007).

**Table 2:** Quotations from focus groups illustrating themes from Fig. 2.

**1. Philosophy of substance misuse treatment and services**
Recovery models and cycle of change
Until the individual user wants to stop and wants to adhere to the treatment programme from inside themselves and not just as a sort of tick box exercise almost, or as going through the motions then no incentive is going to particularly work, it has got to come from within (*Ex-service user group*)
I think that is a really blurred role and this is one of my more fundamental ethical objections with this idea is that we as clinicians should be providing people with information for them to make informed choices, the moment we start offering financial incentives we then undermine our role in doing that, we are saying well actually now we know what's better for you and you start to adopt a more paternalistic attitude (*Multidisciplinary specialist team staff*)
Is this abstinence or harm minimisation, as far as I understand we work towards harm minimisation with the option of abstinence, whereas this would be making quite a large statement about abstinence as the way forward and that is not our decision to make, it is the client's decision (*Multidisciplinary specialist team staff*)
Behavioural model
It does work quite well as part of a behavioural therapy, maybe an ABC sort of type, where you’ve looked at the antecedents of behaviour, I’ll get my benefits, then I want crack, I therefore use crack, the consequences are I feel miserable, … another consequence you could put in to that is “I don’t use I get my voucher” so … it's a behavioural treatment anyway isn’t it? (*Multidisciplinary specialist team staff*)
And it is interesting because there are, dare I say it, doctors who will sit down with a drug rep and be quite happy that they’ve been given a pen and there is something about the symbolic nature of giving something which is of very small monetary value, which is just about some kind of token appreciation (*Specialist addiction psychiatrists*)
Yeah that sort of material support (putting credit in the electricity meter) does make a big difference to any stage of recovery but certainly to the early stages because if you’re giving up something nice, and lets face it we use because it gives us a buzz, if you’re giving that buzz up you want to see some sort of payback, you don’t want to give up using and find that you’re in a freezing flat (*Service user advocacy group*)
Existing forms of coercion/incentives within treatment
I personally think we already have CM but we call it a letter to social services, a letter to the magistrate. How many people come in to services because they don’t want to lose their kids, they don’t want to lose their job or they want to get a flat, you know we already have contingency management (*Service user advocacy group*)
Contingency management as ‘part of a toolkit’
I think it would be really interesting to have this as an option, because we have a number of options at the moment to explore with a patient like this but they’re fairly limited aren’t they, if there is another option which we may have some discomfort about how it would work in our clinic but there is an evidence base and it's a bigger menu to choose from and to discuss with the patient. (*Specialist addiction psychiatrists*)
My fear with all these contingency management programmes is they are (a) substitute for effective treatment and they will become the measure by which you are assessed, (that) it will be used instead of the services providing good motivational techniques, good key working, good care planning, it will simply be how are you doing on your contingency management and that will be the only measure of how you are doing (*Service user advocacy group*)
**2. Practicalities and impact on service provision**
Practicalities of implementation
I just wonder about the initial two pounds for vouchers, whether that's really enough to get a patient to come to the clinic three times a week initially and then the other issue is that (the vignette) says that it keeps increasing so how long do you continue doing it? Are (patients) coming along and will (they) be getting ten pounds or twenty pounds? (*Specialist addiction psychiatrists*)
For three urine samples a week you know the incentive just isn’t there …, given the amount of money I’d be looking at spending on what I was doing. … the monetary value (£2) I wouldn’t get out of bed for, let alone stay sane (*Service user group*)
Contingency management is a complex well thought out psychological and principled matter, how would this package be introduced and withdrawn? How do I introduce this to a client? How do I introduce it with psychological principles? They are rewarded appropriately, that is then withdrawn within the package, appropriately? Those are big questions, not answered in the UK as yet (*Multidisciplinary specialist team staff*)
Opportunity cost
Do we know whether this incentive would attract any extra funding because we don’t have the staff to be offering to see clients (to be urine tested) three times a week? (*Multidisciplinary specialist team staff*)
Equity
I just feel that everyone should be treated equally, and whereas discriminating against somebody, it should be for all or none (*Multidisciplinary specialist team staff*)
I think it should be based on the individual service user as opposed to the mass (*Service users*)
Perverse incentive
If you are trying to target people who were poor at attending and saying “we will reward you to come along”, it is an incentive to all the others to establish themselves as a non-adherer to get on to that scheme (*Multidisciplinary specialist team staff*)
Impact on therapeutic relationship
My other regret is that we spend so much time watching them pee and testing their urines and talking about those urine results is there will be no time left to actually engage with patients, and think about why they use drugs at all (*Specialist addiction psychiatrists*)
It might have actually got me through the door in the early days until those therapeutic alliances were developed and all the motivational stuff was getting me to come back because it was working for me. It would have been a good hook. It wouldn’t have kept me in treatment, I don’t think, but it might have helped me during the early days to actually engage properly (*Service user group*)
I think it cheapens the work that we are actually doing because you know we hope that we are making some input into making people change and that is down to the way (we) work with them, not giving them a prize for coming, giving them a goody bag for turning up, its because of what happens in the intervention when they are seeing us (*Multidisciplinary specialist team staff*)
**3. Wider ethical concerns**
Public perception: fears about increasing stigma towards addicts
I don’t think that anything would be politically acceptable to [*tabloid newspaper*] readers, but giving someone heat and light might be slightly more acceptable than giving them the money to go and score again (*Service user advocacy group*)
Its’ not likely to work in people that have a lot of money, so does that stigmatise the people we are working with because (those) we’re tempting fall into a very poor sub group of society? (*Multidisciplinary specialist team staff*)
If the government feels it is that important in terms of the public health need, to do something different, then let them apply it across the board in terms of incentivising everyone to have the hepatitis immunisation, it's not what we as health workers I think should be doing, but if the government's choosing to do that because they believe that's the only way to address the problem then, that's their choice (*Multidisciplinary specialist team staff*)
Use to which incentive is put (*Alternative incentives to money seen as positive*)
It could help some people, but then a lot of people would just abuse it and come in and like I said it’d go towards the next pipe, so in that sense it will be bad because you’re encouraging them to use rock (*Service user group*)
Societal vs individual factors
It's a totally different scenario (referring to the Hepatitis B vaccination vignette), because it's about other people, it's about the public, it's not about the patient at all, it's because you may be helping the public by immunising this man because you don’t want him to spread hepatitis B, so it's an incentive, it's a public health incentive I think (*Multidisciplinary specialist team staff*)
I can really see the sense of rewarding people to have their vaccinations, and I think that's a win-win situation (*Multidisciplinary specialist team staff*)
when you’re a sex worker … you’ve got kids to think of, they’ve got to go home to their wife, you know, it's just a vicious circle” (*Service user group*)
**4. Evidence of effectiveness**
If the reason why it's been promoted is because of the evidence base ok, it's in America so be it, but nevertheless there is evidence that it works, so from a pragmatic point, our job is to support people to stop using drugs as far as I’m concerned. (*Multidisciplinary specialist team staff*)
Critically might there be nuances that would make what happens in Idaho not completely replicable in Mansfield? (*Specialist addiction psychiatrist*)
Just because something works should we be using it at all? And I think we’ve skipped the first part a bit, you know all sorts of things work but I don’t remember them all being acceptable so, … just because it works doesn’t mean its good to my mind (*Specialist addiction psychiatrist*)
